# Coral Reef Surveillance: Infrared-Sensitive Video Surveillance Technology as a New Tool for Diurnal and Nocturnal Long-Term Field Observations

**DOI:** 10.3390/rs4113346

**Published:** 2012-10-31

**Authors:** Markus Dirnwoeber, Rudolf Machan, Juergen Herler

**Affiliations:** 1 Department of Integrative Zoology, Faculty of Life Sciences, University of Vienna, Althanstrasse 14, A-1090 Vienna, Austria; juergen.herler@univie.ac.at; 2 Department of Neurobiology, Faculty of Life Sciences, University of Vienna, Althanstrasse 14, A-1090 Vienna, Austria; rudolf.machan@univie.ac.at

**Keywords:** coral, monitoring, radio transmission, remote video, unmanned station, 24-h cycles

## Abstract

Direct field observations of fine-scaled biological processes and interactions of the benthic community of corals and associated reef organisms (e.g., feeding, reproduction, mutualistic or agonistic behavior, behavioral responses to changing abiotic factors) usually involve a disturbing intervention. Modern digital camcorders (without inflexible land-or ship-based cable connection) such as the GoPro camera enable undisturbed and unmanned, stationary close-up observations. Such observations, however, are also very time-limited (~3 h) and full 24 h-recordings throughout day and night, including nocturnal observations without artificial daylight illumination, are not possible. Herein we introduce the application of modern standard video surveillance technology with the main objective of providing a tool for monitoring coral reef or other sessile and mobile organisms for periods of 24 h and longer. This system includes nocturnal close-up observations with miniature infrared (IR)-sensitive cameras and separate high-power IR-LEDs. Integrating this easy-to-set up and portable remote-sensing equipment into coral reef research is expected to significantly advance our understanding of fine-scaled biotic processes on coral reefs. Rare events and long-lasting processes can easily be recorded, *in situ*-experiments can be monitored live on land, and nocturnal IR-observations reveal undisturbed behavior. The options and equipment choices in IR-sensitive surveillance technology are numerous and subject to a steadily increasing technical supply and quality at decreasing prices. Accompanied by short video examples, this report introduces a radio-transmission system for simultaneous recordings and real-time monitoring of multiple cameras with synchronized timestamps, and a surface-independent underwater-recording system.

## 1. Introduction

Behavioral and quantitative studies in reef ecosystems have long been based on direct observation and monitoring, e.g., [1]. The basic concept of a diver or snorkeler gaining data by visual census techniques such as transect observations [2] has yielded invaluable information, but was soon recognized to involve a set of drawbacks that affect data quality and quantity [3-6]. Such drawbacks include time and depth limitations, restriction to day-time and less cryptic species, observer influence on animal behavior, intra- and inter-observer reliability as well as the need for well-trained observers or experimentalists who are fit for diving. Accordingly, a range of other methods and non-intrusive technologies have been developed for marine monitoring studies, with increasing emphasis on video techniques. The first attempts to use video systems for observing animal behavior date back over 50 years, e.g., [7,8]. Modern equipment ranges from simple traditional video monitoring using standard camcorders to highly sophisticated autonomous robots.

The introduction of diver-operated video transects [9] increased observer reliability by eliminating the need for highly trained experts in the field as well as other known biases [10], including the “first section effect” or the “edge effect” [11]. Nonetheless, the behavior of fish towards snorkelers, divers or swimmers (attraction, flight) can alter results [12,13] and the abundance of small, cryptic, coral-associated along with nocturnal or timid species tends to be underestimated or even missed [3,9,14]. The use of unmanned video stations to observe aquatic environments eliminates many of these obstacles. Video assessments of fish diversity and abundance, for example, are commonly conducted by remote video stations [15], combined with either stereo video [16] and/or baited video systems [17]. Such assessments by remote video stations and/or diver-operated video transects typically involve short observation periods and therefore benefit from high-definition camcorder equipment [18].

However, high-definition camcorders as used for quantitative and qualitative videometric measurements of size, diversity and abundance of reef organisms [19] are not necessarily always of advantage and applicable under all circumstances, which highlight the need to adopt camera equipment to certain fields of research. In that manner, the development of a special CaveCam [20] was necessary to study small crevices and caves. This invention was a step forward within the related field, leading to significant progress and new insights, e.g., [21-23]. Similarly, field observations intended for behavioral ecologists studying fine-scaled biological processes and interactions of the benthic community of corals and associated reef organisms typically benefit if observation periods can be extended to complete 24 h cycles.

Unmanned video stations are yielding new and unexpected discoveries in modern coral reef ecological studies [24,25]. Such efforts, however, are restricted by standard camcorder observations that are of high quality but short (a few hours) due to their limited power and data storage capacities [26]. Timer-controlled camcorders [27] may extend such observation periods although they do not extend the overall recording time or enable continuous observation. Continuous long-term observation typically involves fixed video stations that are dependent on a land- or ship-based cable connection for power and data storage [28]. While such equipment yields accurate data, it is also characterized by low flexibility while being expensive and difficult to build, transport and handle. Similarly, real-time applications for instant data gathering and life images on land are characterized by a direct connection between submerged video equipment and the associated computer [29].

For behavioral ecologists, the likelihood of observing complex and long-lasting processes, rare events or undisturbed nocturnal activities highly depend on record length and an “invisible” light spectrum that interferes only minimally if at all with natural nocturnal behavior. Fish eyes, for example, are known to be insensitive to long wavelengths (*i.e.*, deep red or infrared light) [30,31]. Although infrared (IR) illumination offers such an “invisible” light spectrum, it was used in behavioral studies to explore nocturnal activity patterns of aquatic organisms by only a few researchers [32-37].

The present study is, to our knowledge, the first to introduce modern standard video surveillance technology as a new tool for coral reef observations of 24 h and longer, including nocturnal recording with IR-light. The constructed system is especially designed for field observations in which entire 24-h cycles are of importance and thus standard camcorders would fail. It is highly flexible, has no land-based cable connection and can be easily set up and relocated by a single diver. The surveillance system is composed of commercially available parts inserted into custom-made housings. Two kinds of such mobile autonomous underwater video surveillance stations are introduced: A surface-independent system with direct-recording underwater and a second system with an emerging antenna and radio-transmission for simultaneous recordings of multiple cameras and live viewing on land.

## 2. System Description

The reef surveillance system is based on combining readily available parts of standard surveillance technology into custom-made waterproof housings. The market in surveillance hard- and software is rapidly expanding, and the state-of-the-art parts we used for constructing the reef surveillance system already have or will soon have follow-up models. Therefore, rather than explaining every system part in detail, this section will explain the principle idea and general parts needed to construct both a radio-transmission system with a surface antenna for simultaneous viewing and recording of multiple cameras in real-time on land as well as a surface-independent, direct-recording system. However, a detailed description including hardware parts and our housing constructions of both systems is provided as supplementary material. Traditionally, research and university institutions have employed technicians who can construct custom-made housings as well as electrical engineers for necessary electronic control circuits.

Each system consists of three main parts: the camera unit, a direct-recording or radio-transmission unit, and a battery pack. An independent IR-light system with two high-power IR-LEDs, supplemented by a battery pack, provides illumination at night and is described as a fourth unit.

### 2.1. Camera Unit

**Figure 1:**
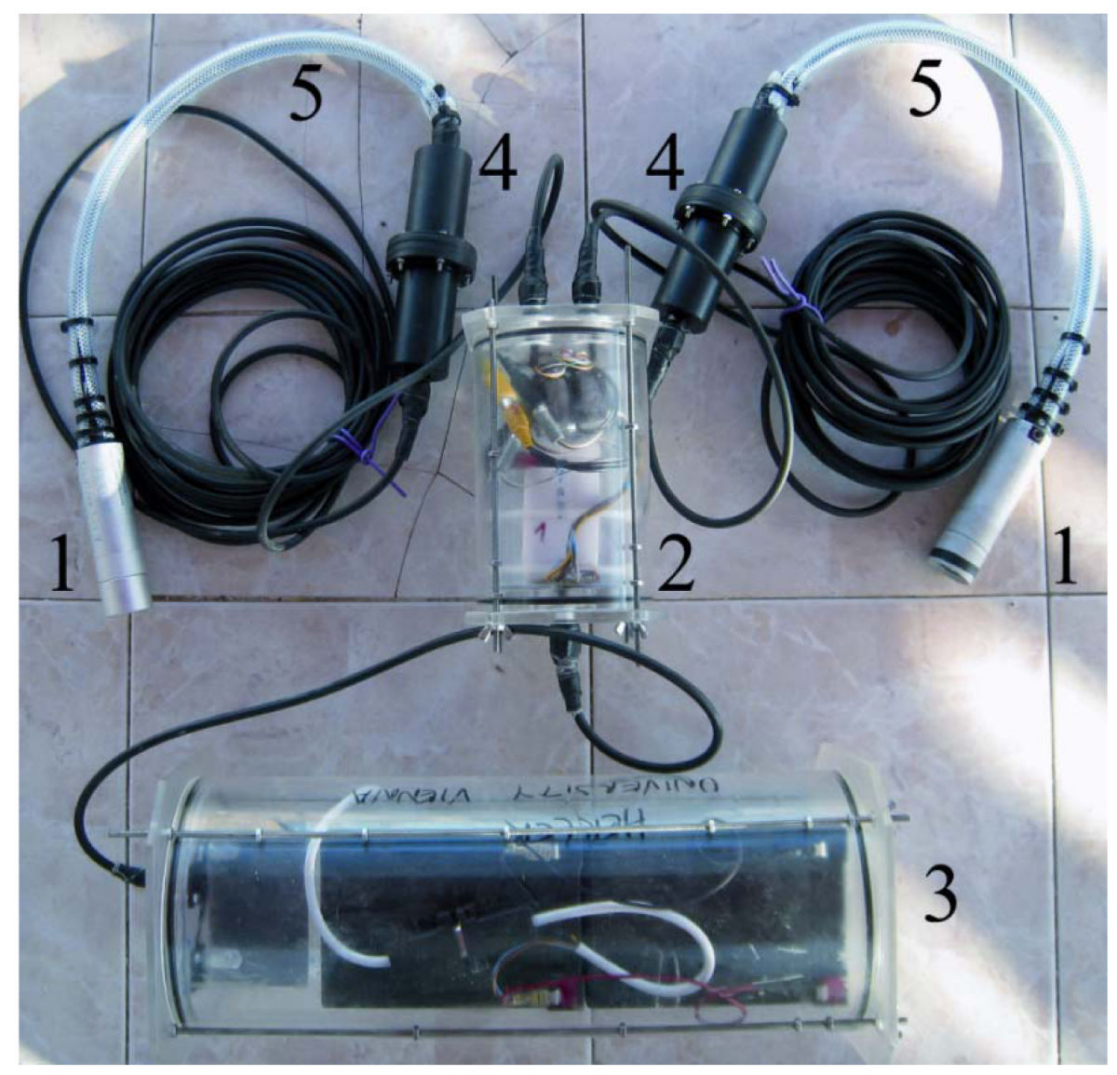
Overview image of the direct-recording underwater video surveillance system. It consists of two cameras (1) (finger cameras in this figure) connected to two single-channel digital video recorders in a separate housing (2) and a double-battery pack (3). In this system the camera cables are interrupted by a waterproof plug connection (4) to disconnect and exchange cameras. The front part of the camera cables has been coated by a flexible tube (5) to minimize mechanical stress through cable deformations.

**Figure 2:**
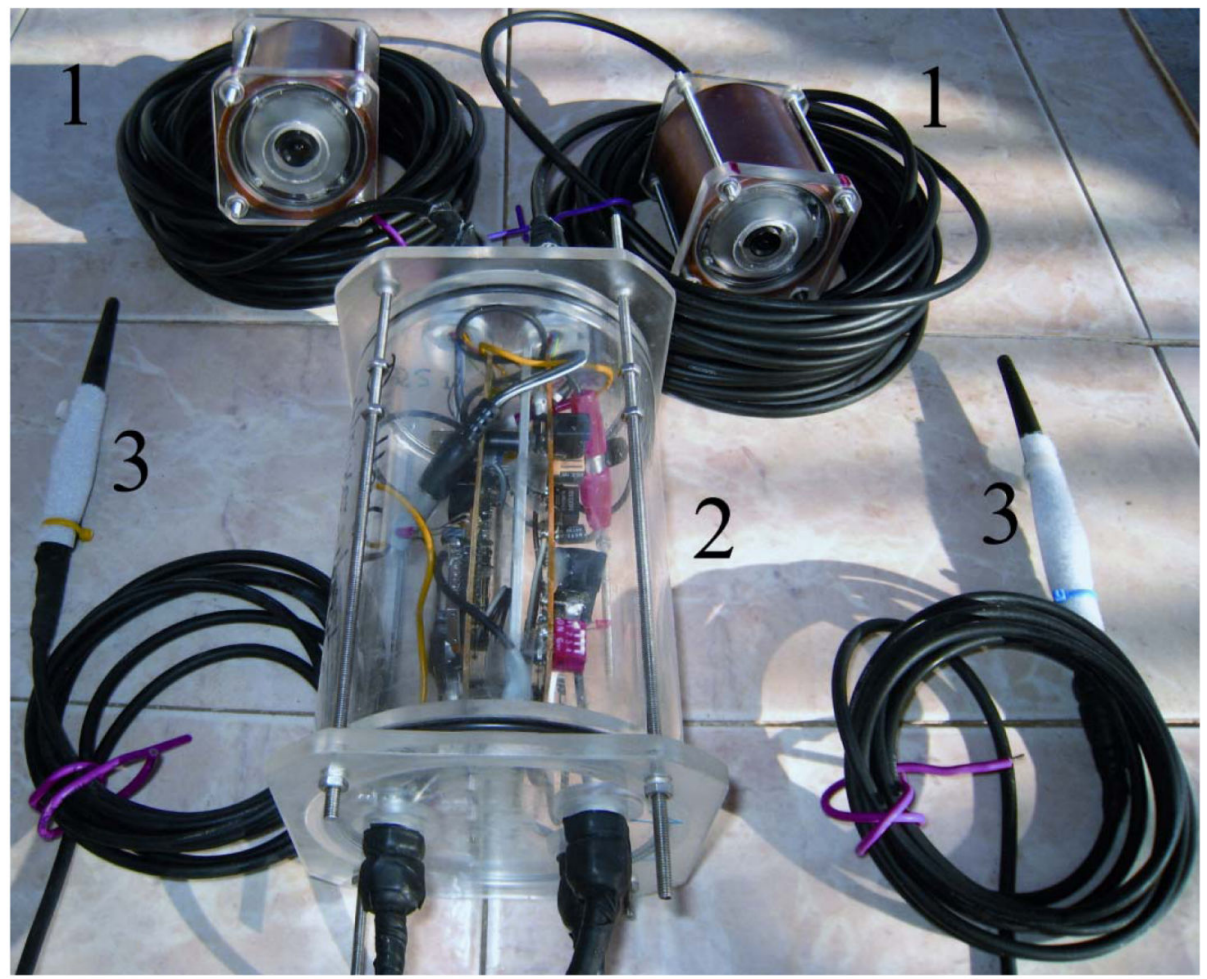
Overview image of the radio-transmission underwater video surveillance system for simultaneous recordings of multiple cameras and live viewing on land. It consists of two cameras (1) (board cameras in this figure) connected to two transmitters in a separate housing (2). The back lid of the housing has two cable glands for the antenna cables (3) and one for the power supply cable. The single-battery pack housing is not shown.

Camera types in surveillance technology are numerous. When applying a camera for reef surveillance it is important to choose highly light- and IR-sensitive, closed-circuit television cameras with digital signal processing, a 12 mm thread for exchangeable lenses and the highest possible resolution (795 × 576 pixels at the time this manuscript was written). These 1/3″ (inch) chip cameras usually have a day (color) and a night (black and white; B/W) mode as well as a multitude of automatic functions (e.g., white balance, back light compensation, gain control). Many camera types are tiny and inconspicuous, which is ideal for a setup in spatially constrained places such as a coral reef. Additionally, cameras are available with an ingress protection (IP) of 68 and are therefore already waterproof and do not require constructing a custom-made housing. The camera is connected to the front lid of either the direct-recording or the radio-transmission unit. Given that cameras in surveillance technology are miniature and low-energy consumers, multiple cameras can be simultaneously connected to these units. Our two camera units consisted of two cameras each, connected by 15 and 30 m of cable (Figures 1 and 2). Cable length can be adjusted to individual needs. Some system parts must be deployed in shallow water (e.g., the transmission unit with its emerging surface antenna) and long camera cables provide more flexibility while the image quality remains unaffected (compared to long antenna cables). As the camera unit is directly connected to the front lid of the direct-recording or radio-transmission unit, we additionally constructed waterproof plugs (Figure 1) that allow changing single cameras (see auxiliary equipment section).

### 2.2. Direct-Recording/Radio-Transmission Unit

These units have identical front lids (camera connection), which allow us to connect different camera units (e.g., with different camera types and/or pre-adjusted camera lenses or settings, cable lengths) to the respective recording and/or transmission unit.

The direct-recording unit (Figure 1) is fitted with either one single-channel micro digital video recorder (DVR) per camera or with a two-channel micro DVR for two cameras at once. Such micro DVRs should be able to record each channel with 25/30 fps (PAL and NTSC compatible) and with the best resolution available on the market (704 × 576 pixels in the system presented here) and must be able to operate with a 32 GB SD-memory card. This will allow storing almost 40 h of real-time video at high quality (1,500 kbps). The DVRs must be set on automatic recording and their timestamps should be synchronized manually (e.g., by setting time and date with a single remote control). The back lid of the custom-made housing is equipped with one cable that connects the direct-recording unit to the battery back.

The radio-transmission unit (Figure 2) is equipped with one 2.4 GHz transmitter per camera. Ideally, front lids and therefore housing dimensions of both the direct-recording unit and the transmission unit are identical for exchanging cameras. In order to fit two transmitters for both cameras into our housing, we fixed the dismantled circuit boards to a central plate connected to the back lid (see supplementary material). Each camera signal is transmitted via a low-loss antenna cable that exits the housing at the back lid together with the cable connection to the battery back. To minimize the quality loss inherent to antenna cables, these cables should be kept as short as possible (2.5 m in our system). The antenna cables have a waterproof connection to 100 mm dipole bar antennas and these must be fixed above the water. Video signals can be received over a distance of up to 5 km with 26 dB parabolic dish antennas, up to 1 km with 14 dB directional Yagi™ antennas, or to about 100 m with 100 mm dipole bar antennas.

On land, one 2.4 GHz receiver per camera signal must be connected to a multi-channel DVR as used in standard video surveillance technology. Similar to the micro DVR, this DVR should have 25/30 fps available for each camera for recordings in real-time; the resolution should be as good as available (720 × 576 pixels in our system). A 1 TB internal hard disc allows a storage capacity of approximately 100 days of non-stop video for two channels in real-time at maximum resolution. Through a monitor the surveillance software allows simultaneous viewing and recording from one to all video signals in either split screen or full screen mode. A portable version of such a receiver-and-recording-system is described below (auxiliary equipment section).

Both DVR systems–the single-channel and the multi-channel DVR–produce manufacturer-specific file formats that can be watched on a PC with the surveillance software included. These systems are ideal for scientific purposes because they are designed for monitoring as well as storing and managing huge amounts of video data. Prescheduled recording times can be assigned to each video signal (e.g., permanent or 15 min of every hour), timestamps facilitate analyses, and each software offers basic tools for searching and browsing through the data archive, watching the videos (simultaneously on the multi-channel DVR) and exporting videos as AVI-files. Files are usually automatically split into smaller portions of either size or time (e.g., 300 MB or 1 h).

### 2.3. Battery Packs

All electronic control circuits (see paragraph: electronic control circuits) are placed inside the battery packs, and a 2-m-long cable connects the battery pack with the respective consumer unit. Should the battery pack be placed on the reef flat, then this length allows changing of batteries on a swimming platform (e.g., an inflatable boat) during low tide. As battery backs are custom-made, the size and number of batteries can be adjusted to the demand of all consumer units in respect to operation times. We used 12 V maintenance-free, sealed lead-acid batteries with a capacity of 12 Ah. Single-battery packs for the radio-transmission system and double-battery packs for the direct-recording system supplied power for more than 24 h.

### 2.4. IR-Light System

**Figure 3:**
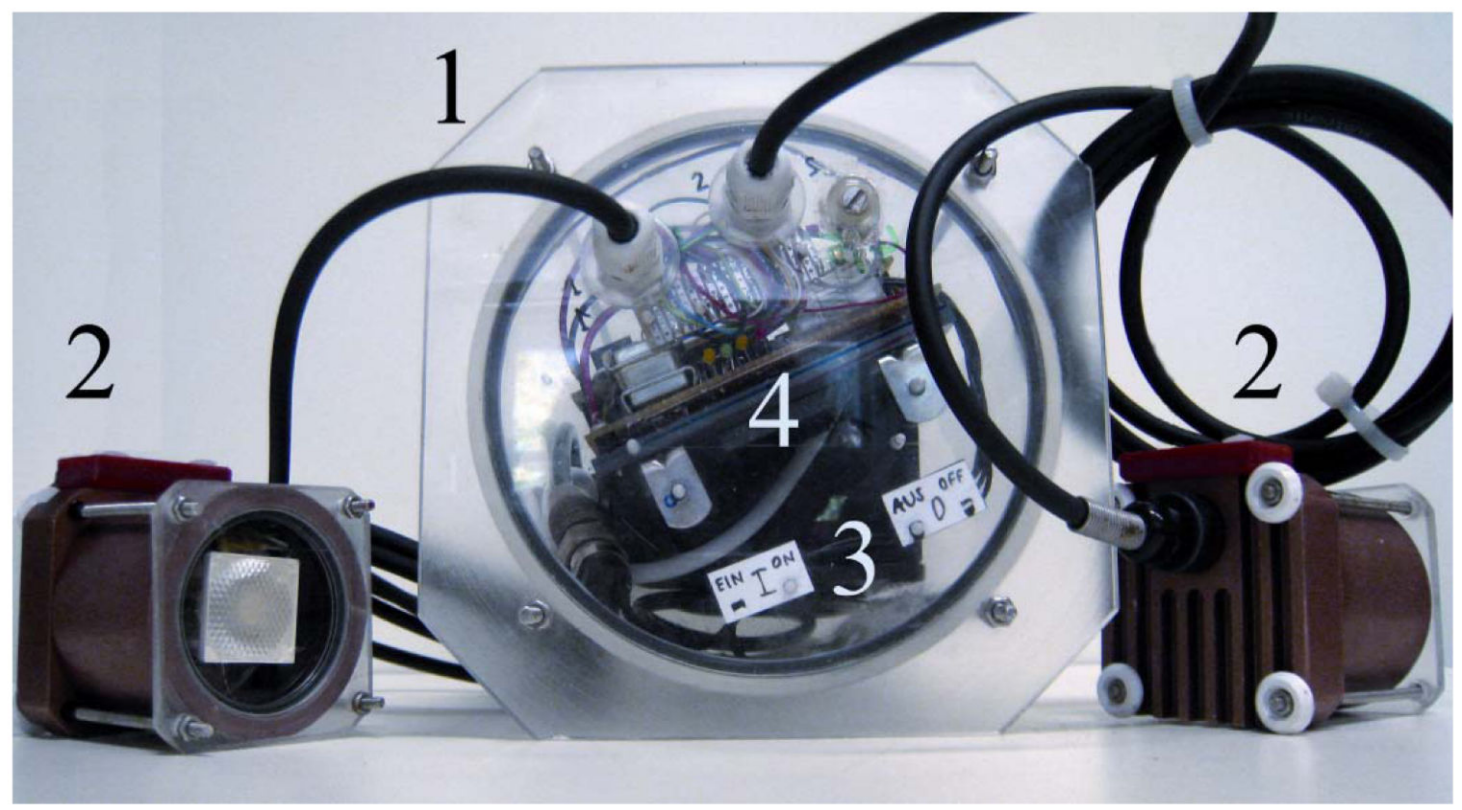
Close up image of the infrared (IR) light system. A single-battery pack (1) is used to supply two IR-LEDs (2) with power for up to 9 h, and a clock timer is used for autonomous operation. The image also shows the on/off magnetic switches (3) and the indicatory LEDs (4) that all battery packs are equipped with (a yellow warning LED indicates low capacity, a green and orange LED indicates activity, and a red LED indicates water ingress in any of the subsystems or cables).

The IR-light system consists of two separate custom-made IR-LED housings connected via two 2-m-long cables to the IR-single-battery pack (Figure 3). Each high-power LED emits a wavelength of about 855 nm and has a 32° divergence angle lens. Compared to the battery packs described above, the single battery pack for IR-LEDs includes additional switches to adjust the power output (300, 500 or 700 mA) for each of the two IR LEDs (up to 9 h at full power with the batteries described above).

### 2.5. Electronic Control Circuits within Battery Packs

Electric functions include main power on/off magnetic switches that can be activated from outside; an optional clock timer; continuous monitoring of battery voltage to prevent damage due to deep discharge (25% of capacity), including a low capacity warning (<90% of capacity) upon starting the system; a water intrusion detection in cables and attached units to prevent damage by electrolysis with an emergency shut-off in case of fault; and a display of malfunctions. The IR-battery pack has an additional current (=intensity) control in three adjustable steps. Further details intended for electronic engineers together with a block diagram of the electronic control circuit can be found in the supplementary material.

### 2.6. Auxiliary Equipment

**Figure 4:**
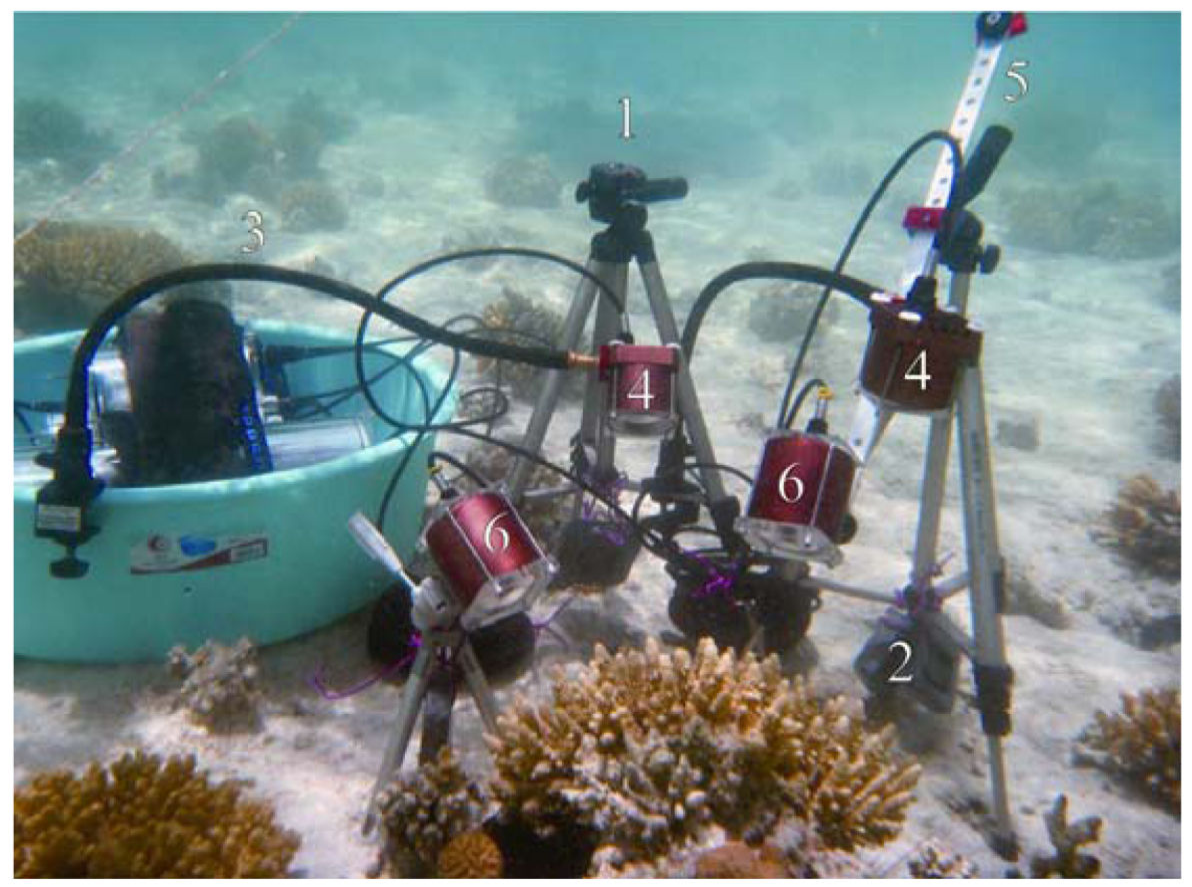
Symbolic underwater image of the video system. Tripods with a 3-way-pan-tilt head (1) and fixed diving weights (2) provide a stable and easy setup of cameras under water, while gooseneck stands (3) orientate the infrared-lights (4). To facilitate alignment of camera distances and for recordings in bird’s eye view, flat aluminum bars (approx. 50 × 1 × 0.5 cm) perforated with 1/4″ holes (5) are used as extensions between cameras (6) and tripods.

A set of accessories was used to facilitate handling and/or widen the applicability:

Tripods with a 3-way-pan-tilt head and fixed diving weights are used to provide a stable and easy setup of cameras under water; gooseneck stands are used to orientate the IR-lights (Figure 4).

To facilitate alignment of camera distances and to record in bird’s eye view, flat aluminum bars perforated with 1/4″ holes are used as extensions between cameras and tripods (Figure 4).

A range of 1/4″ thread camera screw plates fixed to the camera and IR-light housings, and a range of 1/4″ thread camera screws, are used to connect the cameras to the aluminum bars.

To connect/disconnect the cameras from the main cables of the lid of the housings, we constructed a waterproof connector made of PVC and cable glands. This allows us to use the cameras without the lid of the recording/transmission unit for laboratory experiments and is practical when cameras are exchanged. The PVC cable connector is shown in Figure 1 and consists of two hollow pieces that are screwed together and sealed by O-rings. Inside are the cinch and coaxial DC connections.

A variety of filters can be used for specific demands. UV- and IR-cut-off filters used during daylight more reliably reproduce color, while an IR-passing filter used during daylight (blocking the visible light), in combination with the IR-light system, provides better insight into the dark regions of highly bright/dark contrasted areas (e.g., interstices of a branching coral).

We also developed a portable heat-, rain-, and dust-resistant field box for the receivers, the multi-channel DVR and a small LCD monitor (see supplementary material). With such a box, the system can be used in areas without electricity (operated by a car battery) or where the transmission distance would be too large or shielded by structures and other sources of radio-interferences.

## 3. Field of View and Recording Distance

Using auto-focus under water can lead to focusing problems associated with environmental conditions (e.g., drifting particles in front of the lens, focusing on background objects). We therefore used exchangeable lenses with seven different fixed focal lengths (1.8, 2.1, 3.6, 4.3, 6, 8 and 25 mm) and without auto-focus. The lack of auto-focus and zoom requires pre-selecting lenses and recording distances according to object size beforehand on land as well as to focus manually at this distance. Because of the different refraction index of air and water, objects under water appear closer and larger than they actually are. Therefore the recording distance for a given field of view differs between air and water and must be adjusted. The correct recording distance (and focus) under water is obtained by multiplying the distance in focus in air required for a given field of view with the refraction index of water (1.33).

## 4. System Assessment

We used this system mainly for etho-ecological observations of the highly cryptic endofauna of reef-building corals and their interactions with corallivorous fishes as well as to monitor *in situ* caging experiments and experimental aquarium setups. During testing the systems and for preliminary studies, we produced more than 200 h of video footage in a depth of 1 to 8 m. We successfully recorded whole diurnal cycles on a regular basis and the maximum recording periods were 30 h (radio-transmission) and 36 h (direct-recording). Recording distances to objects ranged from 11 cm to 1.5 m and additionally included observation of the open water above the reef with infinite focus. The range of lenses enabled close-up studies of areas of only a few cm2 and the high-resolution finger and board cameras we used enabled us to recognize even small drifting particles. We successfully counted butterflyfish bites taken on single coral colonies throughout several days during *in situ* caging experiments. Therein, wide angle lenses with a focal length of 1.8 mm enabled to record 4 coral colonies at once from a distance of just 60 cm above the substrate (sample video radio-transmission: supplementary video 1; sample video direct-recording: supplementary video 2).

**Figure 5:**
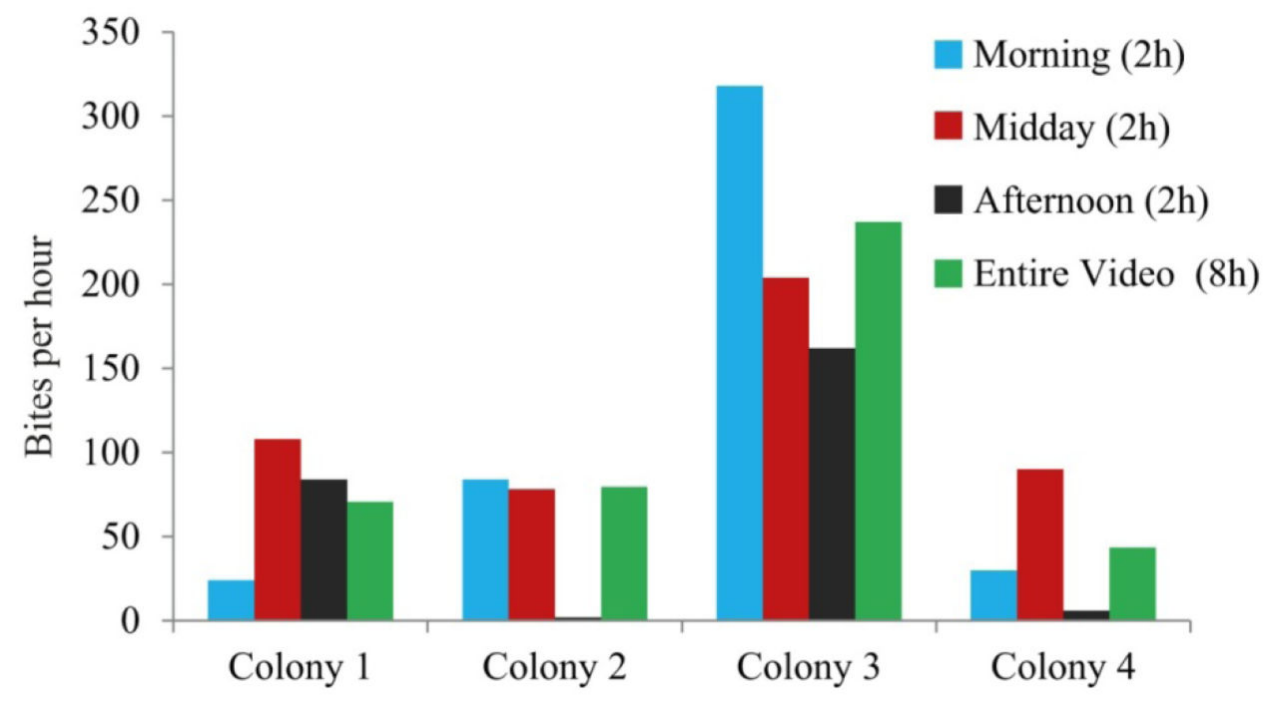
Different bite rates of butterflyfishes on four coral colonies calculated from the same video recording using either short-term intervals of two hours (morning, midday and afternoon) or the entire video sequence of eight hours.

To demonstrate the utility of the system, we used one of the recordings obtained during the *in situ* caging experiment to test whether a prolonged recording period provides more accurate data than typical short-time recordings of standard camcorders. The respective video lasts 8 h and shows butterflyfishes foraging on four different colonies. For this test, the overall recording time of 8 h was subdivided into three 2 h-long intervals (morning, midday and afternoon) and the bites per hour calculated from each of these intervals were compared to the values derived from the overall recording time (Figure 5). This test clearly demonstrates that due to different grazing rates in the course of a day the most accurate value is provided by the long-term monitoring method. Chi-squared tests calculated for the number of bites (standardized to the lowest observed value) between every 2 h-interval and the overall recording reveals that bite rates differed significantly between all short- and the long-term recording (morning *vs.* overall: χ^2^= 20.6, *p* < 0.001; midday *vs.* overall: χ^2^ = 13.4, *p* < 0.01; afternoon *vs.* overall: χ^2^ = 72, *p* < 0.001).

Videos obtained by radio-transmission were easily received over a given distance of approximately 300 m using the parabolic dish antennas. The field box with its dipole bar antennas received clear images over a given distance of approximately 100 m. The water intrusion detection was activated one time as a wrong O-ring dimension led to water ingress into the battery pack. Although the battery pack and inner electronics were submerged in sea water, electrolysis could be prevented by the emergency shut-off. The inner electronics were rinsed with fresh water and continued to work after drying. The preparation of each system averaged 30 min on land and another 30 min for setting up a system under water. In good weather conditions, batteries could be exchanged on an inflatable boat without removing the system from the water.

One of the main novelties of this system is the prolonged observation period which increases the likelihood to observe rare events as well as long-lasting processes. We therefore decided to provide short video clips that demonstrate such events including nocturnal observations with IR-illumination:

Supplementary video 3 shows the obligate coral dwelling fish *Gobiodon histrio* leaving the shelter of its coral colony to catch plankton from the water column. This rare event, which was captured by the direct-recording system during sunrise, has not been documented before and provides a potentially important insight as the feeding behavior of this genus is not totally clear [38,39].

An interesting example for a long-lasting process was captured by chance and only discovered upon watching the recorded video footage. Supplementary video 4 is an edited video of a 6 h long recording in which the coral crab *Tetralia* sp. actively manipulates the corallivorous snail *Drupella* sp. This previously unknown behavior is showing promise for a mutualistic behavior by resource defense that was easily captured by the radio-transmission system. This demonstrates how long observation periods can lead to new discoveries and insights.

Excellent results were obtained for nocturnal observations because coral-dwelling crab carapaces and fish eyes absorb the IR-light and therefore appear black. This leads to very good contrast against the coral, which is highly reflective and appears bright white in IR-light. Supplementary video 5 is a time-lapse video obtained at night by IR-illumination, which shows again a novel and long-lasting nocturnal behavior of coral crabs grazing on (and/or cleaning) coral branches.

A sharp image during daylight may look slightly blurry at night under IR-illumination. This is caused by the different spectrum of infrared *versus* visible light and can be avoided by using focal lengths with higher focal depths (*i.e.*, <4.3 mm focal length) and by keeping the recording distance at a minimum for a desired object width. Alternatively, a camera can be focused as described above but in IR instead of visible light conditions for special nocturnal setups. We also achieved good results by setting the camera on the IR-sensitive B/W mode while focusing in natural daylight. In general, IR-light is quickly absorbed under water and therefore LEDs and cameras should be positioned close to the object and distances exceeding 1 m (combined 2-path-length from the IR-LED to the object and from the object to the camera) should be avoided.

The rapidly evolving market of surveillance and video technology generally promises a continuous increase of video quality at decreasing costs of newer models. However, one drawback is still that surveillance technology is generally designed to store a maximal amount of video footage without the need to watch these data on a regular basis, whereas for scientific purposes all produced videos are intended to be watched and analyzed. Live videos, even from high-resolution cameras, continue to suffer a slight quality loss from video compression upon recording. To ensure a high quality of recorded videos the compression and pixel resolution options of the DVR must be set correctly. With the maximum pixel resolution of 720 × 576 (radio-transmission) and 704 × 576 (direct-recording) the surveillance technology used in our systems will not provide the quality obtained by high-definition camcorders. However, the obtained video quality was more than sufficient for potential analyses of behavioral data of even very small objects (less than 2 cm).

The applicability of the two systems differs according to water depth. Employing the radio-transmission unit means that the transmitting antennas must be above the water surface, whereas the direct-recording unit is surface independent. Although the cameras themselves can be submerged depending on the cable length, the transmitting unit should be in shallow water to minimize the quality loss inherent in antenna cables. This circumstance may limit the range of use depending on local topography and weather conditions. Using radio-transmission to a multi-channel DVR is especially favorable if more than one camera is used to watch the same object as the synchronous recordings and the shared timestamp facilitates parallel video analysis.

Table 1 summarizes the main characteristics of the two systems compared to standard camcorders.

## 5. System Discussion

Video stations for monitoring and studying aquatic organisms have proven to be a useful tool for undisturbed observations, e.g., [40]. Apart from minimizing the time spend underwater, the videos can be re-analyzed whenever necessary, eliminating the need for extensive field training and guaranteeing high observer reliability. Nonetheless, flexible systems that do not require a land- or ship-based cable connection have power and storage restrictions, limiting results to short snapshots of highly complex biological interactions, e.g., [41]. Digital surveillance systems have received a major boost over the last decades with increasing camera resolutions, storage capacities and quality of compressed videos. For long-term observations (>24 h)—including the nocturnal observation through IR-technology—the reef surveillance system introduced here offers an innovative tool for behavioral ecologists and other researchers to study reef organisms in much greater detail. The diurnal and nocturnal recordings help capture long-lasting processes and rare events, boosting the potential for new discoveries and insights.

Although the system can be deployed in both fresh and seawater, it is ideal for observing the shallow environment of a coral reef. The tiny and inconspicuous modern surveillance cameras allow the observation of spatially very restricted areas as they are commonly found in coral reefs. Similarly, it is a powerful tool to observe the cryptic and shy species frequently inhabiting such areas. The system can easily be combined with abiotic sensors to monitor behavioral responses to a changing abiotic environment (e.g., light, temperature, sound, currents/waves, nutrients) as well as with software intended for image based sensing, e.g., [42,43]. By providing extensive observation periods, it is ideal for ethological long-term observations of locally restricted, sessile or little mobile reef organisms such as coral colonies. The nocturnal recordings throughout the night facilitate determining coral spawning times and durations and enable observing the behavior of coral polyps and closely associated organisms during plankton feeding.

The actual area that can be effectively imaged with IR-light depends on the distance that IR-light can travel underwater. The strong absorption of IR-light by water implies that the distance between IR-LED, object and camera is limited. The maximum distance depends on the hardware (e.g., radiant flux, angle of radiation, emitted wave length, light sensitivity of camera) as well as on abiotic factors (e.g., attenuation coefficient of a given wavelength in water with specific optical constituents). Furthermore, as light travels a two-path-length from the IR-source to the substrate and from the substrate to the camera, the properties of the substrate also define optical constituents such as absorption and reflection, thus affecting the amount of light perceived by the camera. Similarly, drift load in the water body such as plankton also reduces the amount of light due to scattering. Taken together, these variables prohibit the calculation of a maximum distance for nocturnal recordings applicable under all circumstances and settings. Theoretical calculations for illuminating and recording (two-path-length of equal distance) a white object in clear water using the hardware and output data of our system (see supplementary materials) and with a 10-fold higher light intensity arriving at the camera than the minimum sensitivity of the camera (0.05 lux) is 1.05 m for each path. This calculated maximum should be handled with caution for the above reasons. Especially in shallow coastal areas such as coral reefs drift load can be high. As mentioned in the assessment section, empirical tests clearly showed that recordings of a combined two-path-length of approximately one meter gave very good results for recording highly reflective coral colonies. Combining IR-light with IR-sensitive cameras successfully captures undisturbed nocturnal behavior and therefore offers a great field of possible applications. In experimental studies this system can reveal the processes which lead to particular experimental outcomes rather than merely documenting the start and end situation without using video. Applications therefore extend to the long-term monitoring of any spatially restricted area and may even be a useful tool in reef management, fishery biology and similar fields. Underwater surveillance might be used to assess the local impacts of human activities, fish diversity, fish behavior towards human-induced structures (e.g., artificial reefs, fish facilities, fish ladders) or serve as a tool for education and awareness (e.g., live reef television, internet streaming).

The enormous range of products in surveillance technology allows great flexibility for specific demands. With introducing this system, we want to stimulate new research questions by highlighting the potential for the creation of innovative field studies with this system and encourage researchers to adjust components and functions to their demands. Numerous additional options are available that include global remote access or the combination with pan-tilt-zoom cameras, which can then be remotely controlled from anywhere in the world. Since most components are generally cheap we suggest looking for high quality products upon choosing the digital video recorder model. From our experience in the field and at this point of time, this is the crucial component in standard video surveillance technology that may reduce the quality of live images. Yet, the fast advancement in digital video technology promises increasing quality and decreasing costs of portable 12 V (or lower) miniature cameras and recorders in the near future.

## 6. Conclusions

The reef surveillance system introduced here is a flexible open-end system for observations in coral reefs or other environments that can easily be setup by a single diver. Although the low transmission of infrared-light through water restricts nocturnal recording distances to approximately one meter, its range of use extends from observing small-scaled structures, habitats and cryptic and/or shy species to larger areas mainly defined by focal lengths and recording distances during daylight. The small size of cameras allows a setup in spatially restricted areas. The key improvement and uniqueness compared to traditional video monitoring is the extensive observation period (24 h and longer) including multiple cameras and the option of viewing radio-transmitted video signals on land in real-time as well as the night-time deployments using inconspicuous infrared-light. Through prolonged recording periods we could show that obtained data are more accurate compared to short-time recordings. Furthermore, the provided video clips demonstrate that significant knowledge advancements and new findings can be expected by using this system, as it is capable of capturing both long-lasting processes and rare events.

Compared to the pixel resolution of normal high-definition camcorders (1,920 × 1,080), the system we introduced herein (see supplementary material section) performs at a lower quality (720 × 576/704 × 576). Nevertheless, this video quality was more than sufficient to observe and analyze organisms in the field (see video clips). Furthermore, the continuously developing market in surveillance technology already provides hardware parts (camera and recorder) that are capable of also producing high-definition videos. Compared to conventional camcorders, this system is additionally preferable due to the inherent nature of surveillance technology in being a tool specifically designed for continuous monitoring and observation. A huge amount of data can be stored, browsed and handled easily. Recording intervals can be pre-selected for each camera individually and for any given time and/or day interval on a daily, monthly or even yearly schedule. The option of combining multiple cameras with a shared timestamp including the possibility of watching these videos in real-time on land is similarly ideal for scientific purposes.

Given that lenses and distances are chosen according to object size and that manual focusing takes the refraction index of water into account, there are not many uncertainties/errors remaining upon using this system. The main uncertainties by using the radio-transmission system are possible interference signals caused by the local radio-environment as well as the need to send signals via an antenna on the water surface. Uncertainties caused by the surface dependence are foreseeable and might restrict its use depending on the local topography or weather condition. Interference signals on the other hand are hardly foreseeable, less predictable and might be caused by fixed (e.g., obstacles) and mobile (e.g., boats, mobile phones, walkie-talkies) objects. Such interference signals can be eliminated by increasing the performance of transmitter and/or receiver antennas through using directional antennas and by keeping the transmission distances as short as possible (e.g., by using the portable field box described in the supplementary material section). An uncertainty upon using the surface-independent direct-recording system is the fact that our system did not have an underwater screen to control the alignment of camera and object. Although we never experienced problems upon such alignments, this potential uncertainty can be solved by choosing mini recorders with screens or by integrating a separate on-demand screen into the open-end system.

The whole system is constructed in a way that all electronic consumers can be either operated by battery or by usual 12 V DC power plugs. Therefore the waterproof cameras can be submerged in experimental aquarium setups while running on normal electricity and directly record on the single- or multi- channel digital video recorder. This flexibility makes the reef surveillance system especially attractive for behavioral studies that have to deal with rare events or long-lasting processes that exceed the typical 1.5–3 h recordings of standard camcorders and for researchers who need to collect field and aquarium data throughout 24 h day-and-night video setups.

## Supplementary Material

Video 1

Video 2

Video 3

Video 4

Video 5

Video legends

Supplementary Material

## Figures and Tables

**Table 1:** Overview of the main characteristics and differences between a standard high-definition camcorder and the two reef surveillance systems for radio-transmission (RT) and direct-recording (DR).

Characteristic	Camcorder	RT	DR
Recording time	~3 h	~30 h	~36 h
Infrared sensitivity	Model-dependent	Yes	Yes
Open end system	No	Yes	Yes
Multiple cameras	No	Yes	Yes
Synchronized timestamp &	No	Yes	No
simultaneous watching			
Live image on land	No	Yes	No
Surface independent	Yes	No	Yes
Auto-focus	Yes	Lens-dependent	Lens-dependent
Timer control	Model-dependent	Yes	Yes
Video quality	Very high	High-medium	Medium
Pixel resolution	1,920 × 1,080	720 × 576	704 × 576
