# Supplementary material for: Coral Reef Surveillance: Infrared-Sensitive Video Surveillance Technology as a New Tool for Diurnal and Nocturnal Long-Term Field Observations
Source: Remote Sens (Basel). Author manuscript; Available in PMC 2014 May 12. (PMC4017077; doi:10.3390/rs4113346)

Supplementary Information

1. Detailed System Description

Each system consists of three main parts: the camera unit, a direct-recording or radio-transmission unit and a battery pack. An independent infrared (IR)-light system with two high-power IR-LEDs, supplemented by a battery pack, provides illumination at night and is described as a separate unit. All electricity consumers are designed to work on 12 V DC. In order to be able to easily change cameras or use them in a standard lab environment, we decided to stay with readily available standard connectors (cinch for video signals, co-axial DC plug for power).

We also recommend using a transparent material for the construction of housings. This allows to see the inner electronics and LEDs, dirt or sand on any of the O-rings, and to visually control for any water ingress or moisture. We decided to use 4 mm thick acrylic glass upon constructing the battery packs. Although acrylic glass is generally a more fragile and recalcitrant material, this thickness was sufficient so securely operate the reef surveillance system in shallow water. We suggest choosing materials and thickness of housings according to maximum operational depths. Especially the surface independent direct-recording system can be easily submerged to maximum SCUBA diving limits by choosing other materials (e.g., polycarbonate) and/or wall thicknesses.

1.1. Camera Unit

Two types of IR-sensitive closed-circuit television cameras with digital signal processing, a 12 mm thread for exchangeable lenses and a resolution of 795 × 576 pixels are used. The first camera, an ABUS Eyseo® Profiline (TV7043) waterproof finger camera is light-sensitive down to 0.05 lux, switches to black/white mode below 2 lux and is IR-sensitive up to 940 nm. Its small (110 mm length, 32 mm diameter) waterproof housing (IP 68) comes with a 30 m long cable. The manufacturer-sealed front cover must be opened for lens exchange and we used additional O-rings to maintain waterproofness. The second type of camera (VCvision Germany®-VC15850) is a wide dynamic range board camera with an on screen display for setting and adjusting a variety of camera functions. It is more light- and IR- sensitive (0.01 lux, up to 1,100 nm) and the highly light-sensitive color mode (0.1 lux) is equipped with an automatically IR-blocking filter.

The custom-made housing for the board camera consists of an anodized aluminum tube with acrylic glass lids sealed by O-rings (Figure S1(A)). In order to adjust for different lens lengths, the board camera needs to be fixed to the front lid (Figure S1(B)). The back lid was equipped with a S6 Subtronic® cable gland equipped with a 6 mm thick and 15 m long Subtronic® 8-core cable.

1.2. Direct Recording Unit

To directly record videos under water, two AKR-200 single-channel micro digital video recorders (DVR) were fitted into an acrylic glass tube (Figure S2(A,B)). Each DVR is capable of recording videos in 4CIF resolution using H.264 compression in real-time, and a 32 GB SD-memory card stores almost 40 h of real-time video at high quality (1,500 kbps). The DVRs are set on automatic recording, and synchronized timestamps of the two DVRs are simply achieved by placing the devices next to each other and setting time and date with a single remote control. Concerning the AKR-200 we must add that the videos of this single-channel micro-DVR model were sometimes slightly grainy if many

pixels changed simultaneously. Given that this system is used stationary such extensive pixel changes are rather unlikely. However, waves in light-exposed shallow water areas result in light travelling through the recorded picture, which may lead to such pixel changes causing the increased pixelization of videos. This circumstance is due to the processor performance of the micro-DVR that we obtained in combination with its H.264 codec, which calculates such pixel changes. The manufacturer of the single-channel micro DVR that we used for our system has single- and double-channel micro-DVRs that can decrease the compression rate through higher processing capacities, which eliminates such pixelization (e.g., AKR-70T and F1-110). Therefore, we recommend using these follow-up models.

The 2 cm thick acrylic front and back lids are sealed with O-rings and equipped with polyamide IP68 Jacob® PERFECT cable glands. For additional safety against water intrusion, the last thread turn of each cable gland was milled even and replaced by an O-ring and its cab sealed with Loctite® 5331 upon closing. To prevent water intrusion within and between the cable wires in case of a cable break, a short strip of insulation was removed from each of the eight cable wires at different levels (to prevent short circuits between the wires) and sealed with epoxy glue. This cable section was placed inside the cable gland which was then closed and filled up with two-component epoxy glue. Two cables through the front lid connect the cameras to the recorders/transmitters, and one cable through the back lid connects the unit to the battery back.

Figure S1. Board camera unit: (**A**) Overview of the parts for the board camera housing. 1: Anodized aluminum tube (7 × 7 cm) with a 4 mm thick wall and 1/4″ internal screw thread plate; 2: Acrylic back lid (7 × 7 cm – milled out at inside) with cable gland and O-ring groove; 3: Acrylic front lid (7 × 7 cm – milled out at inside) with a 3 mm thick acrylic glass inlay, four camera thread bars and an O-ring groove. The cable running around the thread bars is partially dismantled and used as sensor for water ingress; 4: Quadratic (4.2 cm) board camera; 5: O-rings sealing the lids; 6: The housing is closed with four thread bars on the outside, going through front and back lid. (**B**) Cross section of the board camera housing. To bring the camera lens close to the front, the front lid has a centre hole (20 mm) with an outer (28 mm) boring in which the acrylic front glass is glued. The camera was fixed to the front lid with four thread bars allowing adjustment. O-ring cross sections are indicated as black dots. The water ingress protection and the thread bars at the outside of the housing are not shown. All measurements are in centimeters.

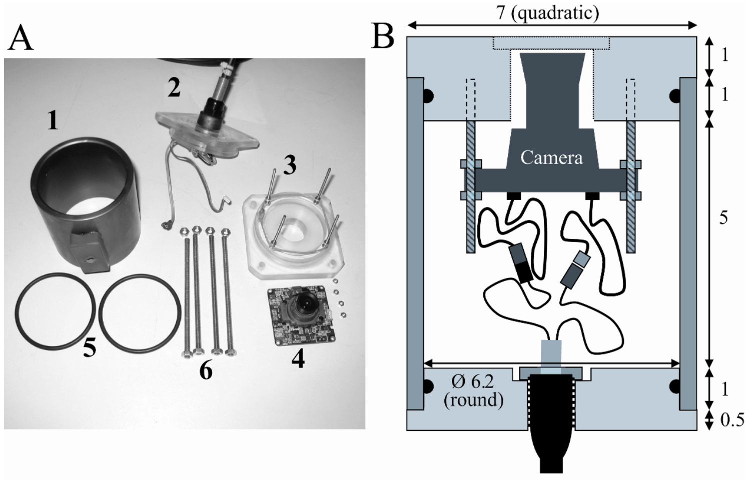

Figure S2. Video recording/transmission unit: (**A**) Schematic drawing of the single-channel DVR housing. The acrylic glass tube has a 5 mm thick wall and is equipped with two DVRs. Measurements for the tube and lids of the DVR housing and of the housing used for radio-transmission are identical. The back lid (left side) of the direct-recording unit differs to the back lid used for radio-transmission unit (depicted in C) by having only one cable exit (to the battery back). All measurements are in centimetres. (**B**) Image of the single-channel DVR housing. Lids are closed with thread bars at the outside corners. (**C**) Schematic drawing of the back lid for the radio-transmission unit, which is equipped with two transmitters and antennas.

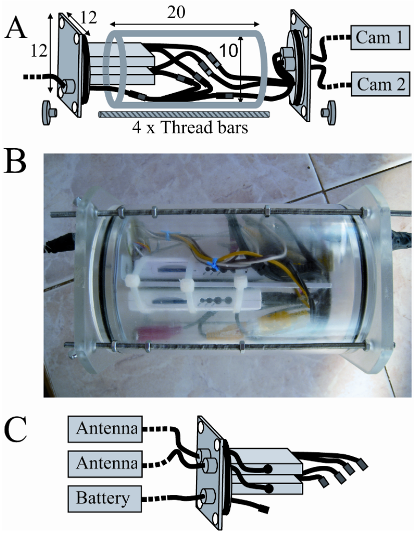

1.3. Radio-Transmission Unit

The same housing was used for the second system with wireless radio-transmission. Since front lids of the direct-recording and radio-transmission systems are identical, the two types of camera units (finger or board camera, with different cable length) can be exchanged as needed. In case of the radio-transmission system, the two cameras entering the tube through the front lid were connected to two 4-channel 2.4 GHz transmitters (DigiSender® DG300), whose dismantled circuit boards were fixed to a central plate connected to the back lid (Figure S2(C)). Channel 1 and 4 were chosen for transmission of the two video signals to ensure minimum interference of signals. Signals were transmitted via two 2.5 m long low-loss antenna cables that exit the tube at the back lid together with the cable connecting to the battery back. The connections between antenna cables and antennas were sealed with self-welding tape and the antennas themselves fixed above the water surface by Velcro® strip to a 2 m long PVC pipe molded into a cement stand. The 2.4 GHz receivers were connected to a multi-channel DVR used in standard video surveillance technology. We used an 8-channel DVR (Neostar JL02) with an MPEG-4 video compression and a 1 TB internal hard disc capable of recording two channels simultaneously in real time (PAL) at a maximum resolution of 720 × 576 pixels for approximately 100 days.

1.4. Battery Packs

Housings consist of acrylic glass tubes, acrylic lids and Jacob® PERFECT cable glands as described above (Figure S3). Since we have been using our systems in the shallow water of a coral reef

(maximum operational depth of 10 m), we chose tubes with 4 mm thick walls to withstand this (relatively low) outside pressure. All electronic control circuits (see paragraph: electronic control circuits) are placed inside the battery packs. A 2 m long Subtronic® cable connects the battery pack with the respective consumer unit. We used 12 V maintenance-free, sealed lead-acid batteries with a capacity of 12 Ah (AEC 12120). Single battery packs are used to supply two video cameras (a total of 180–250 mA) and two radio-transmitters (160 mA) with power for up to 26 h; double battery packs with a combined capacity of about 24 Ah supply two cameras and two single-channel micro-DVRs (250 mA) with power for up to 41 h (limited to 36 h by video storage capacity if set on 25 fps).

Figure S3. Schematic drawing of a single-battery pack. The double-battery pack for the direct-recording system differs by having 38 cm long tubes. All measurements are in centimetres.

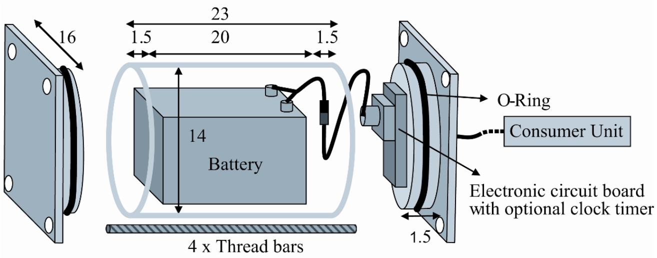

1.5. IR-Light System

The IR-light system consists of two separate custom-made IR-LED housings connected via two 2 m long Subtronic® cables to the IR-(single-)battery pack. Each of the anodised aluminium housings holds a PerkinElmer® high-power LED (Aculed™ VHL-IR1) emitting a wavelength of about 855 nm, and a 32° divergence angle lens system (Aculed™ LHS-AL25-L32) (Figure S4). Compared to the battery packs described above, the single battery pack for IR-LEDs includes additional switches for adjusting the power output (300, 500 or 700 mA) for each of the two IR LEDs (up to 9 h at full power).

1.6. Electronic Control Circuits within Battery Packs

Electric functions include main power on/off magnetic switches that can be activated from outside; an optional clock timer; continuous monitoring of battery voltage to prevent damage due to deep discharge (25% of capacity), including a low capacity warning (<90% of capacity) upon starting the system; a water intrusion detection in cables and attached units to prevent damage by electrolysis with an emergency shut-off in case of fault; and a display of malfunctions. The IR-battery pack has an additional current (=intensity) control in three adjustable steps. The electronic circuits, one per battery housing (Figure S5), were assembled on breadboard. The breadboard was screwed to the lid bearing the off going power-cable, which was soldered directly to the board. On the battery side and for connecting the optional timer we used 9-pin sub-D connectors, which proved reliable and suitable for high-current charging. Leak detection (e.g., to discover invisible cable damage) was accomplished by applying a voltage of +5 V above all other potentials to a screw that has contact with the surrounding water by penetrating the lid and detects any current flow. All connected units were also equipped with inside electrodes, permitting immediate shutdown in case of flooding anywhere in the system. This is

essential to prevent electrolysis responsible for most of the damage to electronics when working in the wet. The circuit was designed for low power consumption (2 mA including status display) using CMOS components, thus enabling it to work for a long time even after low battery switch off. For the cabling between units, if not already part of the device itself, we used an 8-core underwater cable readily available from Subtronic®. Because of the short lengths involved, no special requirements as to signal or power degradation had to be met. Our main selection criteria were handling and robustness in a harsh environment. As we only needed a maximum of four cores, the remaining ones were connected in parallel or saved as spares.

Figure S4. IR-light system: (**A**) Image of the IR-LED housing. 1: Acrylic glass front lid; 2: Anodised aluminium tube; 3: Anodised aluminium back lid with heat sink grooves and a 1/4″ internal screw thread plate on the opposite side; 4: S6 Subtronic® cable gland; 5: Thread bar. (**B**) Cross section of the IR-LED housing. The housing consists of a quadratic acrylic front lid and an anodised aluminium back lid-both milled out at the inside and sealed with O-rings (indicated by black dots)-and an anodised aluminium tube with a 4 mm thick wall. The LED is mounted on the back lid, which also serves as a heat sink. The water ingress sensor and the thread bars at the outside of the housing are not shown. All measurements are in centimetres.

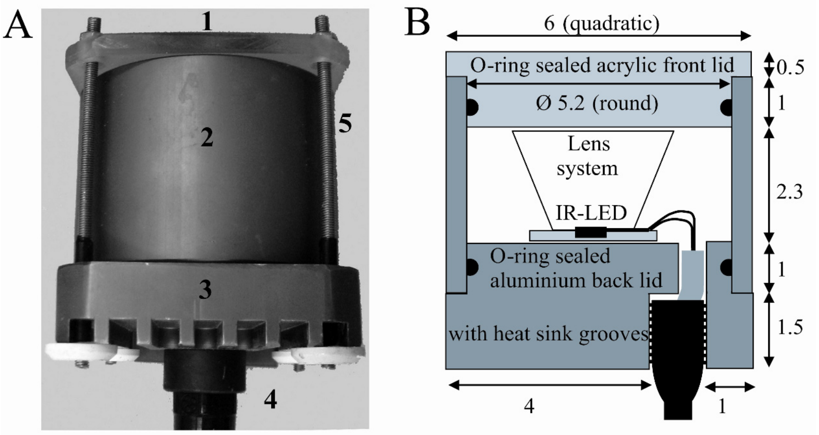

Figure S5. Block diagram of electronic control circuit within battery packs. Stippled line schematically indicates housing and solid line indicates front lid of battery housing.

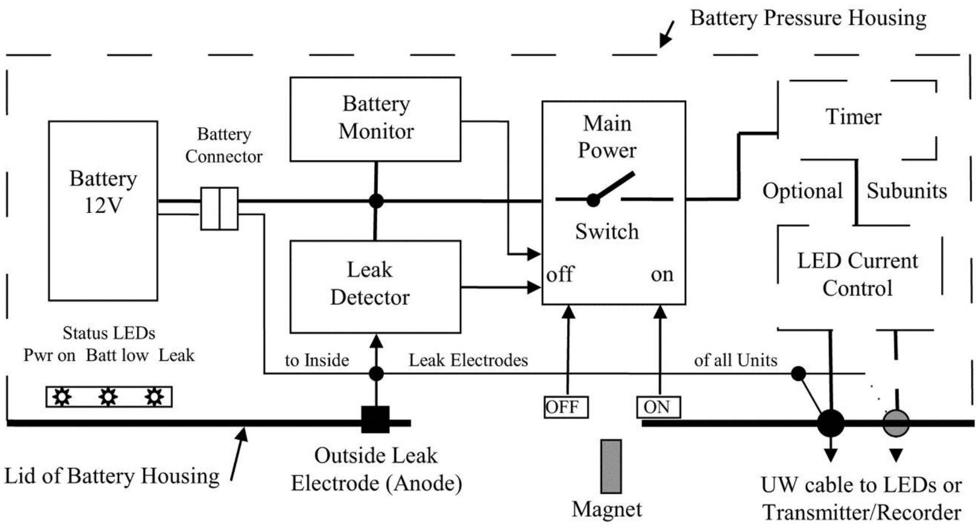

1.7. Portable Field Box

A critical but non-technical aspect of obtained video quality is radio-interference while using the radio-transmission system. Since transmission power is usually regulated and limited by national laws we cannot provide a general recommendation for how to avoid such interferences apart from trying to keep the distance between transmitter and receiver at a minimum. Interferences may or may not occur regardless of the frequency bandwidth used for transmission and mostly depend on the local radio noise. Although we successfully received our signals over several hundred meters by using parabolic dish antennas–which definitely yielded the best reception and may exclude a major part of radio noise–very good results were also obtained by placing a portable field box (even when equipped with 100 mm dipole antennas only) onto the shore close to the transmitting antennas. The portable heat-, rain- and dust-resistance field box can be used for receiving and recording videos by radio-transmission in order to minimize transmission distances or in areas without electricity (Figure S6).

Figure S6. Image collection of the field box. (**A**) We fixed the multi-channel digital video recorder (DVR) to the bottom of a plastic box. The box is ventilated through a standard CPU ventilator (right side) and a few rows of air-inlet holes which are protected against dust intake by micro fibre (left side). (**B**) Two longitudinal bars fixed to the side walls of the box above the DVR support the two receivers (antenna cables exit the box through holes) and a switch box used for power distribution and battery exchange. This switchbox (with a LED-light for indicating battery capacity) enabled the interruption-free exchange of car batteries by reducing the current between new and old battery during switching. (**C**) The plastic box was covered by self-adhesive heat-reflecting isolation foil and a small monitor allowed instant control of the received video signals. (**D**) A standard car battery (12 V/65 Ah) is used to supply the field box with electricity for up to 16 h. Normal 100 mm dipole antennas received the video signals over a distance of 100 m.

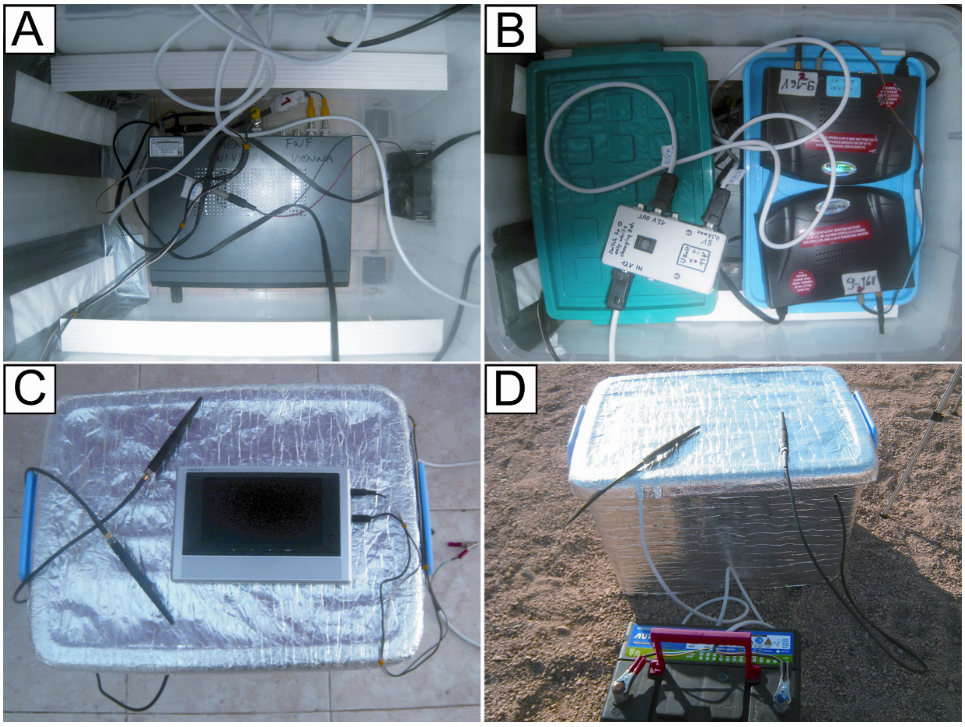

Supplement: Supplementary Material [file NIHMS56088-supplement-01.pdf]
